# Combined polyphenols in *Psidium guajava*-citrus limon leaf extract attenuate fructose-induced cardiac injury by modulating metabolic and oxidative stress pathways in rats

**DOI:** 10.1371/journal.pone.0339641

**Published:** 2026-01-21

**Authors:** Hend A. Essa, Dina E. ElMosbah, Heba A. Fahmy

**Affiliations:** 1 Nutrition and Food Sciences Department, Food Industries and Nutrition Research Institute, National Research Centre, Cairo, Egypt; 2 Department of Pathology, Faculty of Veterinary Medicine, Cairo University, Giza, Egypt; 3 Department of Pharmacognosy, Faculty of Pharmacy, Modern University for Technology and Information, Cairo, Egypt; Emory University School of Medicine, UNITED STATES OF AMERICA

## Abstract

**Background:**

High fructose consumption is associated with metabolic and cardiovascular dysfunction. Polyphenol-rich lemon and guava leaves may protect the heart.

**Objective:**

This study investigated the protective effects of *Citrus limon* leaf extracts, individually and in combination with *Psidium guajava*, against high fructose-induced cardiac injury in rats. We hypothesized that the polyphenol-rich extracts would attenuate cardiac damage through metabolic and oxidative stress modulation, with synergistic enhancement in the combined treatment.

**Methods:**

The chemical composition of lemon and guava leaves was analyzed using LC-ESI-MS/MS, along with total phenolic, flavonoid, and antioxidant assays. Male Sprague-Dawley rats (n = 6/group) were divided into: a control group, a fructose group (20% w/v in drinking water for 6 weeks), and two treatment groups receiving either lemon leaf extract (200 mg/kg/day) or a lemon-guava leaf extract (200 mg/kg/day, 1:1) for 2 weeks, followed by co-administration with fructose for 6 weeks. Blood glucose, insulin, lipid profiles, troponin I, creatine kinases, oxidative stress markers, and inflammatory markers were measured. Histopathological and immunohistochemical analyses were performed.

**Results:**

Sixteen phenolic compounds were identified, with the combined extract showing higher phenolic and flavonoid content and antioxidant activity. Both extracts significantly (p ≤ 0.05) improved insulin resistance and dyslipidemia, significantly (p ≤ 0.05) reduced troponin I and creatine kinase-myocardial band levels, and significantly (p ≤ 0.05) enhanced cardiac antioxidant activity by increasing glutathione, glutathione peroxidase, superoxide dismutase, and catalase, while decreasing malondialdehyde, interleukin-6, and tumour necrosis factor-α. The lemon-guava extract upregulated SIRT1 and NRF2 expression, demonstrating superior efficacy in mitigating fructose-induced cardiac stress.

**Conclusion:**

The combined lemon-guava leaf extract alleviates fructose-induced cardiac stress, demonstrating potential as a plant-based therapeutic agent for metabolic and cardiovascular protection.

## 1. Introduction

Over recent decades, fructose consumption has risen significantly, despite warnings from governments and health organizations regarding the risks associated with high intake of simple sugars. The World Health Organization recommends that free sugars should account for less than 5% of total daily energy intake for optimal health benefits [1]. Many studies support a link between fructose intake and various metabolic dysfunctions associated with metabolic syndrome (MetS), including obesity, increased adiposity, dyslipidemia, insulin resistance, type 2 diabetes mellitus (T2DM), and cardiometabolic diseases [2].

Cardiovascular disease is the leading cause of death worldwide, with well-established risk factors including hypertension, obesity, and other metabolic diseases that can lead to clinical manifestations such as myocardial infarction and pathological cardiac hypertrophy [3]. High intake of fructose and fats can trigger MetS, insulin resistance, and inflammation, which are known to promote cardiac hypertrophy and fibrosis [4]. This inflammatory response is believed to stem primarily from increased oxidative stress within cells, which occurs when there is an overproduction of free radicals or when antioxidant defenses are insufficient. Fructose metabolism generates free radicals that can damage cells and trigger inflammation, making intracellular molecules that respond to antioxidant substances critical for reducing oxidative damage [5]. Silent information regulator 1 (SIRT1) plays a crucial role in these protective mechanisms that respond to cellular damage [6]. SIRT1 activates nuclear factor erythroid 2-related factor 2 (Nrf2), which stimulates the production of protective enzymes like glutathione peroxidase (GPx) and superoxide dismutase (SOD) via the antioxidant response element [7].

Plants are a major natural source of numerous bioactive compounds [8]. For centuries, plant-based preparations have been an integral part traditional medicine, and they continue to be a key component of the contemporary pharmaceutical, nutraceutical, and cosmetic industries. Leaves, which are often discarded as agricultural by-products, are particularly rich in many bioactive molecules and secondary metabolites. The various phytochemical compositions and biological activities of leaf extracts from numerous species have been clarified by recent studies [8–10]. For instance, guava (*Psidium guajava* Linn.), from the Myrtaceae family, is widely recognized for its medicinal and nutritional properties and is cultivated in tropical regions like India, Indonesia, Pakistan, Bangladesh, and South America. Different parts of the plant, including the roots, leaves, bark, stem, and fruit, have long been used in traditional medicine for conditions such as stomachache, diabetes, and diarrhea. Guava leaves are also noted for their antispasmodic, cough-suppressant, anti-inflammatory, antioxidant, and antidiabetic properties [11]. Moreover, Animal studies have confirmed that guava leaf isolates exhibit potent antitumor, anticancer, and cytotoxic effects against certain cancer cell lines [12,13].

Similarly, lemon (*Citrus limon*), member of family Rutaceae, is native to tropical and subtropical Southeast Asia and northern India and nowadays it is widely grown in countries like Italy, Turkey, the United States, Brazil, Mexico, Iran, Argentina, Spain, and China [14,15]. Every part of the lemon plant exhibits antioxidant properties and immune-enhancing effects. Its therapeutic effects are primiraly due to its phytochemicals, such as flavonoids, alkaloids, and phenols, distributed throughout the plant [15]. The bioactive compounds in lemon are associated with a range of health benefits, including antioxidant and anti-inflammatory properties, and have been studied for their potential in managing conditions such as diabetes, hypercholesterolemia, and hypertension [16].

Given the established antioxidant and anti-inflammatory properties of guava (*Psidium guajava L*.) and lemon (*Citrus limon L*.) leaves, we hypothesized that their bioactive compounds might mitigate cardiac damage induced by high fructose intake. This study aims to evaluate the prophylactic effects of these leaf extracts on cardiac metabolic and oxidative stress caused by high fructose consumption. To our knowledge, this is the first study to investigate the combined effects of guava and lemon leaves on fructose-induced cardiac injury in rats. By exploring the activation of the SIRT1/NRF2 pathway and oxidative-inflammatory modulation, we propose this novel plant combination as a natural therapeutic strategy to protect against high fructose-induced cardiac damage. This study not only advances our understanding of plant-based interventions for metabolic and cardiovascular diseases but also highlights the potential of agricultural by-products, such as leaves, as sustainable sources of bioactive compounds for therapeutic applications, offering a sustainable and natural solution to address the global burden of metabolic and cardiovascular diseases.

The study was conducted in two phases. First, a chemical investigation of guava leaves, lemon leaves, and their combination was performed, including assessments of total phenolic content, total flavonoids, antioxidant activity, and Liquid chromatography–electrospray ionization–tandem mass spectrometry (LC-ESI-MS/MS) analysis. Subsequently, the in vitro results informed an in vivo experiment where rats were pre-treated for two weeks with either lemon leaf extract or a guava-lemon leaf combination prior to and during fructose challenge. This prophylactic protocol was designed to evaluate the protective potential of these extracts against fructose-induced metabolic disturbances and cardiac injury in rats.

## 2. Materials and methods

### 2.1. Materials

#### 2.1.1. Plant material.

*Psidium guajava L*.(local name: guava) and *Citrus limon* (L.) Osbeck (local name: lemon) were collected from a garden in El-Beheira, Egypt. They were authenticated by Therese Labib, consultant of Plant Taxonomy, Ministry of Agriculture, Mazhar Botanic Garden, Giza, Egypt. The voucher specimens (M272 and M273, respectively) were deposited by Prof. Dr. Mona Mohamed Marzouk at the herbarium of the National Research Centre (CAIRC).

#### 2.1.2. Chemicals.

DPPH (2–2’diphenyl-1-picrylhydrazyl), catechin, gallic acid, chlorogenic acid, epicatechin, ellagic acid, caffeic acid, coumaric acid, ferulic acid, syringic acid, 3,4-dihydroxybenzoic acid, cinnamic acid, rosmarinic acid, methyl gallate, rutin, quercetin, naringenin, hesperetin, apigenin, daidzein, resveratrol, and kaempferol were purchased from Sigma-Aldrich Co., (St. Louis, MO, USA). For HPLC analysis, acetonitrile and methanol of HPLC grade were utilized. All other utilized chemicals were of analytical grade.

### 2.2. Methods

#### 2.2.1. In vitro study.

**2.2.1.1. *Leaves extraction:*** Air-dried leaves (200 g) were finely ground and subjected to exhaustive extraction using 500 mL of 70% ethanol (three successive extractions). The combined ethanolic extracts were concentrated under reduced pressure at 40°C, yielding 6.5 g and 7.2 g of crude extract for lemon and guava leaves, respectively. The dried extracts were stored in a desiccator for subsequent analyses.

**2.2.1.2. *Total phenolic content (TPC) determination:*** TPC was assessed according to Singleton et al. [17] and Chang et al. [18]. The reaction mixture containing 25 μL extract, 10 μL 10% aluminum chloride, 10 μL 1M potassium acetate, 75 μL ethanol, and 140 μL distilled water was incubated for 30 minutes in the dark. Absorbance was measured at 415 nm (n = 3) using a microplate spectrophotometer. TPC was expressed as mg quercetin equivalents per gram dry weight (mg QE/g DW) (S1 Fig).

**2.2.1.3. *Total flavonoid content (TFC) determination:*** TFC was assessed according to Chang et al. [18]. The reaction mixture containing 25 μL extract, 10 μL 10% aluminum chloride, 10 μL 1M potassium acetate, 75 μL ethanol, and 140 μL distilled water was incubated for 30 minutes in the dark. Absorbance was measured at 415 nm (n = 3) using a microplate spectrophotometer. TFC was expressed as mg quercetin equivalents per gram dry weight (mg QE/g DW) (S2 Fig).

**2.2.1.4. *DPPH radical scavenging activity:*** The 2,2-Diphenyl-1-picrylhydrazyl (DPPH) radical scavenging ability was measured using the Brand-Williams et al., (1995) [19] method. DPPH methanol solution (1.0 milliliter, 0.1 mM) was used to dissolve the methanolic extracts at ambient temperature. The absorbance was measured using a spectrophotometer (UV-Vis-160–1 PC, Japan) at 515 nm after 30 minutes of incubation. The following equation was used to express the results as percentage inhibition:


$$\%\;\text{inhibition}=({\text{A}}_{\text{blank}}\;-\;\frac{{\text{A}}_{\text{sample}}}{{\text{A}}_{\text{blank}}})\;\times \text{100}$$

Trolox was used to create the standard curve, and the results were reported as mM Trolox equivalents (mM TE/g sample).

**2.2.1.5. *Ferric reducing power scavenging activity (FRAP):*** The reducing capacity was determined following Aiyegoro and Okoh [20]. Each extract (1 mL) was mixed with 2.5 mL each of 1% potassium ferricyanide and phosphate buffer (0.5M, pH 6.6), then incubated at 50°C for 20 minutes. The reaction was terminated with 2.5 mL 10% TCA, followed by centrifugation (2600 × g, 10 minutes). The supernatant (2.5 mL) was mixed with 0.5 mL 0.1% FeCl_3_, and absorbance was measured at 700 nm after 10 minutes. Higher absorbance values indicated greater reducing power. Results were quantified using a Trolox standard curve (mM TE/g).

**2.2.1.6. *LC-ESI-MS/MS-MRM Analysis:*** Liquid chromatography-electrospray ionization–tandem mass spectrometry (LC-ESI-MS/MS) was used to analyze and identify the phenolic and flavonoid compounds in lemon and guava leaf extracts. Exion LC™ AC system (USA) was utilized for separation coupled to a detector of SCIEX Triple Quad 5500 + MS/MS system (Singapore) equipped with an electrospray ionization (ESI).

### Multiple-reaction monitoring (MRM) in the positive and negative modes

Using the gradient elution technique, the separation was carried out using a Poroshell 120 EC-C18 column (3.0 × 100 mm, 2.7 µm). Two eluents, A: 0.1% formic acid in water and B: acetonitrile (LC grade), made up the mobile phase and were programmed as depicted in **Table 1**.

**Table 1:** The mobile phase program used for LC-ESI MS/MS MRM characterization of polyphenols of lemon and guava leaf extracts.

Time (min)	%B	Flow rate (mL/min)
0-4	8	0.5
4-12	15	0.4
12-20	20	0.4
20-25	30	0.4
25-25.01	45	0.4
25.01-28	8	0.5

The sample injection volume was 5 µl, and the temperature was maintained at 40°C. MRM in the positive and negative ionization modes was employed in the same run. The mass spectrometer parameters were adjusted as: curtain gas of 25 psi, source temperature of 400°C, ion spray voltage of −4500 for the negative mode and 4500 for the positive one, and ion source gas of 55 psi utilizing MRM parameters as listed in S1 Table.

#### 2.2.2. In vivo study.

**2.2.2.1. *Animals, and humane endpoint:*** The experiment was conducted using healthy adult male Sprague Dawley rats (150–170 g, 4–5 weeks old). The rats were obtained from the animal facility of the Faculty of Veterinary Medicine in Egypt. Before the experiment, the rats underwent a two-week acclimatization period in the animal laboratory under standard laboratory conditions. During experimental procedures, rats were housed individually in a stainless steel cage under conventional settings, with a controlled temperature of 21 ± 2°C, a relative humidity of 55–60%, and a 12-hour light/dark cycle. Throughout the study, the rats were provided with a standard laboratory diet and had free access to tap water.

All necessary precautions were implemented to reduce animal distress, adhering to the principles outlined by the National Center for the Replacement, Refinement, and Reduction of Animal Research (NC3Rs) and complying with the Animal Research Reporting of *In Vivo* Experiments (ARRIVE) guidelines. Daily monitoring was conducted to assess signs of pain or distress, such as weight loss, immobility, and labored breathing, and every effort was undertaken to minimize suffering. No animals required premature euthanasia during the study.

Upon completion of the experiments (8 weeks), all animals were anesthetized via intramuscular injection of 87 mg/kg ketamine and 13 mg/kg xylazine to ensure minimal discomfort. Euthanasia was subsequently performed through cervical dislocation while the animals were under anesthesia.

**2.2.2.2. *Ethical statement:*** This study received ethical approval from the Institutional Animal Care and Use Committee (IACUC) of the Faculty of Veterinary Medicine, Cairo University, Egypt (Approval Number: Vet CU131020241051). The research protocol adhered to the regulations and guidelines established by the National Institutes of Health (NIH) for the care and use of laboratory animals [21]. All experiments were conducted in accordance with relevant guidelines and regulations, including the ARRIVE guidelines (PLoS Biol 8(6), e1000412, 2010).

**2.2.2.3. *Diet composition***: The AIN-93 balanced diet was composed of 58.5% maize starch, 5% cellulose, 3.5% AIN-93 salt, 10% corn oil, 10% sucrose, and 12% casein as a protein source. This diet was supplemented with 1% AIN-93 vitamin mixture and 3.5% AIN-93 salt, following the formulation described by [22].

**2.2.2.4. *Experimental design:*** A total of 24 male Sprague Dawley rats were randomly assigned into four groups (n = 6 per group) as follows:

**Group 1 (Normal Control Group)** Rats had daily access to tap water and a balanced diet for eight weeks.

**Group 2** **(High-Fructose Group)** For the first two weeks, these rats received tap water. Following that, they were given drinking water with a 20% (w/v) fructose mixture for six weeks [23,24]. A balanced diet was provided daily throughout the entire experiment..

**Group 3 (High-Fructose + Lemon Leaf Extract Group)** Rats were orally administered lemon leaf extract (200 mg/kg/day) for eight weeks. Fructose (20%) was introduced into the drinking water after the first two weeks. A balanced diet was provided daily for the entire duration of the experiment.

**Group 4 (High-Fructose + Lemon and Guava Leaf Extract Group)** Rats were orally administered a combination of lemon and guava leaf extracts (200 mg/kg/day in a 1:1 ratio) for eight weeks. Fructose (20%) was introduced into the drinking water after the first two weeks. A balanced diet was provided daily for the entire study duration.Throughout the experiment, all rats had continuous access to their designated diet and water ad libitum.

Body Weight and Food Intake Measurement: Food and water intake were monitored regularly, and body weight was recorded weekly. At the end of the study, the final body weight, body weight gain, and total food intake were calculated. Additionally, the feed efficiency ratio (body weight gain/total food intake) and the Heart Weight Index (HWI), HWI = heart weight (mg)/ body weight (g), were calculated [25].

**2.2.2.5. *Blood and Tissue Sampling:*** Following the 8-week experimental duration, all animals underwent a 12-hour fasting period before euthanasia. Anesthesia was induced with an intramuscular injection of 87 mg/kg ketamine and 13 mg/kg xylazine, preceding sacrifice by cervical dislocation. Blood samples were then collected from the retro-orbital plexus. Sera were subsequently isolated via centrifugation at 3000 rpm for 15 minutes at 4°C (Laborezentrifugen, 2k15, Sigma, Germany) and stored at −20°C until further biochemical analyses.

The heart was immediately dissected, washed in ice-cold saline, blotted dry, and weighed. It was then divided into two portions. One portion was preserved in 10% (v/v) neutral buffered formalin for subsequent histological and immunohistochemical analyses. The second portion, consisting of one gram of heart tissue, was homogenized in ice-cold phosphate-buffered saline (pH 7.4) to create a 10% w/v homogenate using an MPW-120 tissue homogenizer (BitLab Medical Instruments, Poland). The homogenized tissue was then centrifuged at 4000 rpm for 10 minutes at 4°C using a Laboratory Centrifuge 2K15 (Sigma Co., Germany) [26]. The resulting supernatant was collected and stored at −80°C for later biochemical analyses.

**2.2.2.6. *Cardiac, and metabolic parameters Assessment:*** Serum cardiac marker enzyme activities and metabolic parameters, including creatine kinase (CK) and creatine kinase-myocardial band (CK-MB), were assessed using colorimetric and kinetic assay kits, following the International Federation of Clinical Chemistry (IFCC) methods [27]. Fasting blood glucose concentration was determined using the glucose oxidase method [28]. The lipid profile was analyzed by measuring total cholesterol (TC) [29], high-density lipoprotein cholesterol (HDL-C) [30], low-density lipoprotein cholesterol (LDL-C) [31], and triglycerides (TG) [32]. Very low-density lipoprotein cholesterol (VLDL-C) was calculated by dividing the TG value by 5, while non-HDL-C was determined by subtracting HDL-C from total cholesterol. The TC/HDL-C ratio was used as an indicator of cardiac risk, whereas the atherogenic index (AI = LDL/HDL) served as a marker of atherogenicity and ischemic disease risk [33]. All assays were performed using commercial kits from Salucea Co., Netherlands.

Serum Troponin I levels were quantified using a rat-specific enzyme-linked immunosorbent assay (ELISA) kit (Catalog No. SL0713Ra, Sunlong Biotechnology Co., LTD HangZhou, China), in accordance with the manufacturer’s guidelines. Similarly, serum insulin levels were measured using a rat-specific ELISA kit (Catalog No. SL0373Ra, Sunlong Biotechnology Co., LTD HangZhou, China). Insulin resistance was evaluated using the homeostasis model assessment for insulin resistance (HOMA-IR) according to the following formula: HOMA-IR index = [fasting glucose (mg/dL) × fasting insulin (µU/mL)/ 405] as described by [34].

**2.2.2.7. *Assessment of Antioxidant and Oxidative Stress Biomarkers in Heart Tissue:*** The levels of malondialdehyde (MDA), superoxide dismutase (SOD), catalase (CAT), reduced glutathione (GSH), and glutathione peroxidase (GPx) were determined spectrophotometrically following the methods described by Nair and Turner [35]; Sun, [36]; Luck, [37]; Jollow, [38]; and Rotruck, [39]. Biochemical analyses were conducted using reagent kits from Spectrum Diagnostics Company (Cairo, Egypt), following the manufacturer’s instructions. The optical density of all parameters was measured using a Shimadzu UV-2401 PC spectrophotometer (Australia).

**2.2.2.8. *Assessment of Tumor Necrosis Factor (TNF-α) and interleukin −6 (IL-6) in Heart Tissue:*** The concentrations of TNF-α and IL-6 in heart homogenates were measured using rat-specific ELISA Kits (Catalog No. SL0722Ra for TNF-α and SL0411Ra for IL-6, Sunlong Biotechnology Co., LTD HangZhou, China). These analyses were performed following the manufacturer’s instructions, utilizing the sandwich ELISA method and spectrophotometric detection.

**2.2.2.9. *Histopathological examination*:** The heart specimens from each group were collected and fixed in 10% neutral buffered formalin. After processing through graded ethanol and xylene, the specimens were embedded in paraffin and sectioned to a thickness of 3–5 µm. The sections were stained with hematoxylin and eosin (H&E) and examined under a light microscope (DM4B; Leica, Germany) [40].

**2.2.2.10. Immunohistochemistry (IHC):** Immunohistochemical analysis of NRF2 and SIRT1 in the myocardium of heart tissues was performed. Following deparaffinization and rehydration, antigen retrieval was conducted using heat in a microwave. Primary antibodies for NRF2 (16396–1-AP, Proteintech, Germany) and SIRT1 (13161–1-AP, Proteintech, Germany) were applied to the slides at a dilution of 1:200. The slides were then treated with H2O2 for blocking, followed by incubation with a horseradish peroxidase-labeled secondary detection kit (BSB-0015, BioSB, USA). Finally, the slides were counterstained with hematoxylin. Positive staining was quantified as the mean area percentage measured in high-power microscopic fields using ImageJ software.

#### 2.2.3. Statistical analysis.

Statistical analyses were performed using SPSS version 25 and GraphPad Prism 8.4.3. Data are presented as the mean ± standard error of the mean (SEM). Significant differences among experimental groups were determined using a one-way analysis of variance (ANOVA). Post-hoc comparisons were conducted using Duncan’s test. A p-value of ≤ 0.05 was considered statistically significant. Pearson’s linear correlations were also performed to assess relationships between variables.

## 3. Results

### In Vitro

#### TPC, TFC, and antioxidant potential.

**Fig 1:**
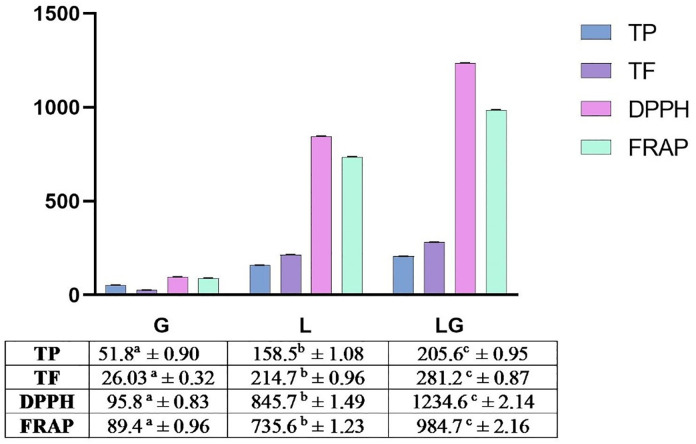
Total phenolic (mg GAE/g DW), flavonoid (mg QE/g DW)contents and antioxidant capacity assays (DPPH, FRAP) of guava (G) and lemon (L) leaf extracts. DPPH and FRAP are evaluated as mM TE/g DW. The measurements are expressed as mean ± SD (n = 3). For each of the examined parameter, a, b, and c denotes that they are significantly different at p ≤ 0.05; DPPH, 2,2-Diphenyl-1-picrylhydrazyl; FRAP, Ferric reducing antioxidant power; TFC,Total flavonoid content; TPC, total phenolic content.

The total phenolic and flavonoid contents, along with the antioxidant assay (DPPH and FRAP) results are depicted in **Fig 1**. The results demonstrated that lemon leaves significantly surpass those of guava in total phenolic and flavonoid contents and also in their antioxidant potential using the FRAP and DPPH assays with an obvious augmentation in their mixture as depicted in **Fig 1**. Pearson correlation (Table 2) showed that there is a significant positive correlation between the TPC, TFC, and the antioxidant capacity assessed by DPPH and FRAP.

**Table 2:** Pearson correlation of the phenolic, flavonoid contents and antioxidant capacities in lemon and guava extracts and their mixture (1:1).

Variables	DPPH	FRAP
TPC	0.9992*	0.9995*
TFC	0.9961	0.9998*

* denotes a significant correlation at p ≤ 0.05; TPC, total phenolic content; TFC,Total flavonoid content.

### LC-ESI-MS/MS- MRM analysis

Phenolics and flavonoids were annotated and quantified in lemon and guava leaf extracts using LC-ESI-MS/MS. Identification was carried out depending on comparison with the standard compounds’ retention times, parent ions (Q1), and product ions (Q3), and previous literature. The LC-ESI-MS/MS chromatograms in MRM mode of 20 standard phenolics and flavonoids are displayed in S3 Fig, while those of lemon and guava extracts are depicted in S4 Fig. Sixteen compounds were annotated, *viz.* gallic acid, caffeic acid, rutin, coumaric acid, naringenin, quercetin, ellagic acid, 3,4-Dihydroxybenzoic acid, hesperetin, cinnamic acid, methyl gallate, kaempferol, ferulic acid, syringic acid, catechin, and chlorogenic acid as represented in **Table 3**. Rutin, the flavonoid glycoside, was detected at exceptionally high concentration in lemon, whereas guava was rich in phenolic acids, *viz.,* ellagic acid, gallic acid, 3,4-dihydroxybenzoic acid, coumaric acid, catechin, besides the flavonoids naringenin and quercetin.

**Table 3:** Identified phenolics and flavonoid concentrations expressed as µg/g dry extract using LC–ESI-MS/MS.

Name	Rt (min)	L	G
		Conc. (μg/g)	
Gallic acid	1.67	7.82	1103.90
Caffeic acid	5.83	ND	9.96
Rutin	9.13	726.84	4.75
Coumaric acid	7.7	59.32	114.80
Naringenin	20.98	373.95	832.93
Quercetin	18.16	5.72	656.83
Ellagic acid	8.97	4.63	1587.56
3,4-Dihydroxybenzoic acid	3.13	31.74	184.15
Hesperetin	22.62	14.29	ND
Cinnamic acid	18.16	22.87	ND
Methyl gallate	5.04	ND	17.74
Kaempferol	22.08	ND	7.85
Ferulic acid	8.89	28.47	5.41
Syringic acid	6.23	15.36	14.38
Apigenin	21.47	ND	ND
Catechin	5.05	ND	938.54
Daidzein	16.22	ND	ND
Chlorogenic acid	5.1	6.86	4.86
Resveratrol	14.27	ND	ND
Rosmarinic acid	13.42	ND	ND

Rt, retention time; L, lemon leaf extract; G, Guava leaf extract; ND, not determined.

### In Vivo

**Based on our in vitro findings, guava leaves alone showed significantly lower antioxidant activity compared to lemon or the combination (** **Fig 1** **); therefore, we focused on the enhanced effect of the combination (guava-lemon extract) in the in vivo model. Lemon-guava leaves improved nutritional parameters, metabolic dysfunction, cardiac biomarkers, oxidative stress, and inflammatory markers in the studied groups of rats.**

The results in Table 4 show that, compared to the normal control group, the high-fructose group exhibited significant reductions (p ≤ 0.05) in final body weight, body weight gain, and feed efficiency ratio. Conversely, this group showed a significant increase (p ≤ 0.05) in water intake, heart weight, and heart weight index. Both the high-fructose + lemon leaves extract group and the high-fructose + guava-lemon leaves extract group significantly improved these parameters, with no significant differences in total food intake (p > 0.05) among all groups. Water intake was significantly elevated in the high-fructose group (p ≤ 0.05 vs. the normal control group), and both treated groups showed intermediate values.

**Table 4:** Nutritional parameters, and heart index of different experimental groups.

Parameters	NC	HF	HF-L	HF-GL
Initial body weight (g)	166.2 ± 4.71^**a**^	166.3 ± 3.66^**a**^	166.4 ± 3.74^**a**^	166.1 ± 3.89^**a**^
Final body weight (g)	260 ± 5.78^**a**^	210 ± 4.52^**b**^	225 ± 3.46^**a**^	236 ± 3.21^**a**^
Body weight gain (g)	94 ± 3.23^**a**^	44 ± 3.61^**b**^	59 ± 3.18^**c**^	70 ± 3.11^**d**^
Total food intake (g)	780 ± 26.42^**a**^	620 ± 23.23^**a**^	669 ± 25.47^**a**^	698 ± 24.81^**a**^
Feed efficiency ratio	0.12 ± 0.01^**a**^	0.07 ± 0.01^**b**^	0.09 ± 0.01^**c**^	0.10 ± 0.01^a^
Water Intake (ml/day)	16 ± 2.54^**a**^	22.85 ± 3.89^**b**^	20.28 ± 4.01^ab^	18.68 ± 3.42^**a**^
Heart weight (g)	0.84 ± 0.06^**a**^	0.89 ± 0.07^**b**^	0.87 ± 0.06^**c**^	0.85 ± 0.06^**d**^
Heart weight index	0.32 ± 0.01^**a**^	0.42 ± 0.02^**b**^	0.39 ± 0.02^**c**^	0.36 ± 0.02^**d**^

NC = Normal Control group, HF = high fructose group, HF-L = high fructose+lemon leaves extract group, HF-GL = high fructose+ guava-lemon leaves extract group. In the same row: the similar letters mean non-significant difference within groups at P ≤ 0.05.

Cardiac stress biomarkers were significantly elevated in the high-fructose group compared to the normal control group (p ≤ 0.05), including creatine kinase (CK), creatine kinase-myocardial band (CK-MB), and troponin I (Table 5). Treatment with lemon leaves extract significantly attenuated these increases (p ≤ 0.05). The combined guava and lemon leaves extract demonstrated superior efficacy, producing the most substantial reductions in all measured cardiac biomarkers compared to the high-fructose group (Table 5).

**Table 5:** Effects of Lemon Leaves and Guava-Lemon Leaves Extract on Cardiac Biomarkers of High Fructose in the different studied groups in rats.

Parameters	NC	HF	HF-L	HF-GL
CK (U/L)	44.30 ± 1.85^**a**^	48.05 ± 0.99^**b**^	46.3 ± 1.75^**c**^	44.92 ± 1.66^**ac**^
CK-MB (U/L)	48.11 ± 1.54^**a**^	101.25 ± 2.45^**b**^	67.84 ± 1.48^**c**^	54.34 ± 1.14^**a**^
Troponin I (µg/L)	0.043 ± .001^**a**^	0.082 ± .003^**b**^	0.070 ± .002^**c**^	0.054 ± .002^**a**^

NC = Normal Control group, HF = high fructose group, HF-L = high fructose+lemon leaves extract group, HF-GL = high fructose+ guava-lemon leaves extract group. CK: creatine kinase. In the same row: CK-MB: creatine kinase-myocardial band, the similar letters mean non-significant difference within groups at P ≤ 0.05.

As shown in Table 6, the high-fructose diet induced significant metabolic disruptions (p ≤ 0.05), characterized by substantial increases in glucose, insulin, insulin resistance, and atherogenic lipid parameters (total cholesterol, triglycerides, LDL-C, and VLDL-C), alongside a significant decrease in HDL-C compared to the normal control group.

**Table 6:** Impact of Lemon Leaves Extract and Combined Guava-Lemon Leaves Extract on blood Glucose, insulin, and Lipid Profile in the different studied groups of rats.

Parameters	NC	HF	HF-L	HF-GL
Glucose(mg/dl)	74.43 ± 1.99^**a**^	231.68 ± 2.31^**b**^	104.35 ± 1.14^**c**^	91.14 ± 0.89^**d**^
Insulin (mU/L)	15.9 ± 0.61^**a**^	20.04 ± 0.84^**b**^	18.73 ± 0.80^**c**^	16.66 ± 0.72^**a**^
IR	2.92 ± 0.99^**a**^	11.46 ± 2.13^**b**^	4.82 ± 1.11^**c**^	3.74 ± 0.97^**ac**^
TC (mg/dL)	68.87 ± 1.22^**a**^	200.9 ± 2.45^**b**^	142.7 ± 1.84^**c**^	115.4 ± 1.80^**d**^
TG (mg/dL)	78.6 ± 1.31^**a**^	302.8 ± 2.84^**b**^	136.05 ± 1.25^**c**^	106.74 ± 1.23^**d**^
HDL-C (mg/dL)	42.57 ± 0.85^**a**^	25.87 ± 0.66^**b**^	30.55 ± 0.68^**c**^	37.10 ± 0.67^**ac**^
LDL-C (mg/dL)	17.21 ± 0.15^**a**^	106.39 ± 1.10^**b**^	80.44 ± 0.86^**c**^	67.79 ± 0.83^**d**^
VLDL-C (mg/dL)	15.72 ± 0.12^**a**^	60.65 ± 0.58^**b**^	27.21 ± 0.22^**c**^	21.34 ± 0.21^**d**^
Non-HDL-C	26.30 ± 0.82^**a**^	175.03 ± 1.15^**b**^	109.70 ± 0.98^**c**^	78.30 ± 0.85^**d**^
TC/HDL-C	1.62 ± 0.02^**a**^	7.77 ± 0.06^**b**^	4.67 ± 0.04^**c**^	1.82 ± 0.04^**a**^
LDL-C/HDL-C	0.36 ± 0.01^**a**^	4.11 ± 0.05^**b**^	2.63 ± 0.02^**c**^	2.35 ± 0.02^**d**^

NC = Normal Control group, HF = high fructose group, HF-L = high fructose+lemon leaves extract group, HF-GL = high fructose-guava and lemon leaves extract group. TC: total cholesterol, HDL-C: high-density lipoprotein cholesterol, LDL-C: low-density lipoprotein cholesterol, TG: triglycerides IR: insulin resistance. In the same row: the similar letters mean non-significant difference within groups at P ≤ 0.05.

Treatment with lemon leaves extract significantly mitigated these alterations (p ≤ 0.05), leading to notable reductions in all measured glycemic and lipid parameters relative to the high-fructose group.

The combined guava-lemon leaves extract demonstrated the most pronounced therapeutic effect, resulting in the greatest significant improvements across all glycemic indices and the lipid profile, effectively reversing the metabolic disturbances induced by the high-fructose diet (p ≤ 0.05).

As detailed in Table 7, cardiac tissue from the high-fructose group exhibited a state of significant oxidative stress and inflammation (p ≤ 0.05), marked by a substantial increase in malondialdehyde (MDA) and pro-inflammatory cytokines (TNF-α and IL-6), alongside a pronounced depletion of key antioxidant defenses (glutathione, glutathione peroxidase, superoxide dismutase, and catalase) compared to the normal control group.

**Table 7:** Effects of Lemon Leaves and Guava-Lemon Leaves Extract on high fructose-induced Oxidative stress and inflammatory markers in the cardiac tissue of the different studied groups.

Parameters	NC	HF	HF-L	HF-GL
MDA (nmol/g tissue)	17.5 ± 1.28^**a**^	47.25 ± 0.86^**b**^	31.65 ± 1.11^**c**^	22.15 ± 0.99^**a**^
GSH (mg/g tissue)	96.65 ± 3.89^**a**^	37.79 ± 1.10^**b**^	73.90 ± 2.54^**c**^	92.56 ± 2.87^**a**^
GPx (IU/g tissue)	27.2 ± 1.99^**a**^	13.10 ± 0.35^**b**^	18.61 ± 0.47^**c**^	24.24 ± 0.64^**a**^
SOD (u/g tissue)	88.6 ± 3.59^**a**^	33.82 ± 0.54^**b**^	68.93 ± 2.53^**c**^	82.34 ± 2.99^**a**^
catalase (U/g tissue)	21.2 ± 2.12^**a**^	8.83 ± 0.73^**b**^	12.42 ± 1.06^**c**^	17.42 ± 1.24^**a**^
TNF-α (pg/mg protein)	32.78 ± 2.17^**a**^	78.67 ± 2.23^**b**^	49.82 ± 1.94^**c**^	38.96 ± 1.16^**a**^
IL-6 (pg/mg protein)	36.85 ± 1.99^**a**^	86.59 ± 2.04^**b**^	54.73 ± 1.57^**c**^	41.86 ± 0.95^**a**^

NC = Normal Control group, HF = high fructose group, HF-L = high fructose+lemon leaves extract group, HF-GL = high fructose+ guava and lemon leaves extract group. In the same row: the similar letters mean non-significant difference within groups at P ≤ 0.05.

Treatment with lemon leaves extract significantly ameliorated this imbalance (p ≤ 0.05), effectively reducing MDA and pro-inflammatory cytokines while restoring the levels of all measured antioxidants relative to the high-fructose group.

The combined guava-lemon leaves extract demonstrated superior efficacy, normalizing the oxidative and inflammatory milieu to the greatest extent. This group showed the most significant reduction in MDA and pro-inflammatory cytokines and the most robust upregulation of antioxidant enzyme activities compared to the high-fructose group (p ≤ 0.05).

### Histopathology

**Fig 2:**
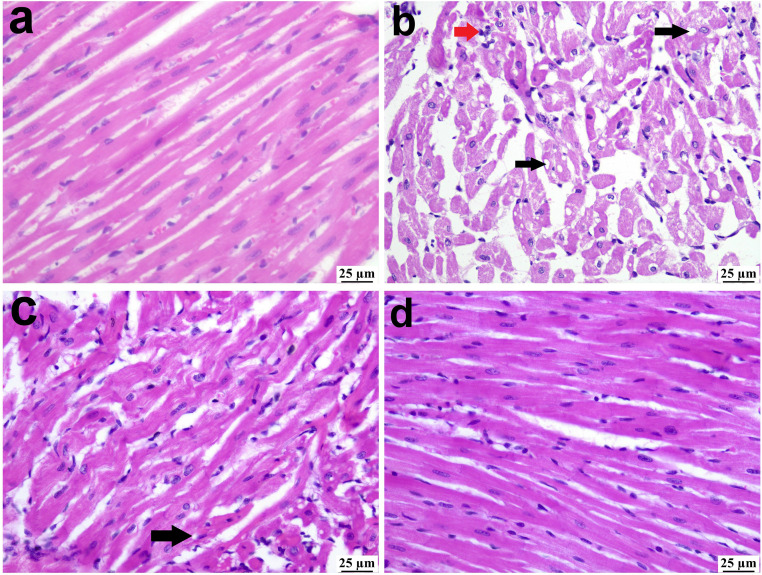
Photomicrograph of rat heart among different groups (H&E stain); (a) normal control group, (b) multiple cardiomyocyte vacuolation (black arrow) admixed with inflammatory cells (red arrow) and necrosis (arrowhead) in HF group, (c) presence of micro vacuoles (arrow) with few inflammatory cells in HF-L group, (d) apparently normal histological structure in HF-GL group.

Microscopic examination of heart tissues revealed a normal histological structure in the normal control group (Fig 2a). In contrast, heart tissues from rats fed a high-fructose diet exhibited marked vacuolar degeneration, characterized by multiple round, variable-sized clear vacuoles within cardiomyocytes, occasionally coalescing into larger vacuoles. Additionally, multifocal areas of mononuclear inflammatory cell infiltration and confluent regions of necrosis in individual or small groups of cardiomyocytes were observed, along with separation of muscle fibers (Fig 2b). Heart sections from the HF-L group showed mild vacuolation and limited inflammatory cell infiltration (Fig 2c). A more pronounced improvement was observed in the HF-GL group, which maintained normal myocardial cellular integrity, with only mild inflammatory cells occasionally seen between muscle bundles (Fig 2d).

### Immunohistochemistry of NRF2 and SIRT1

**Fig 3:**
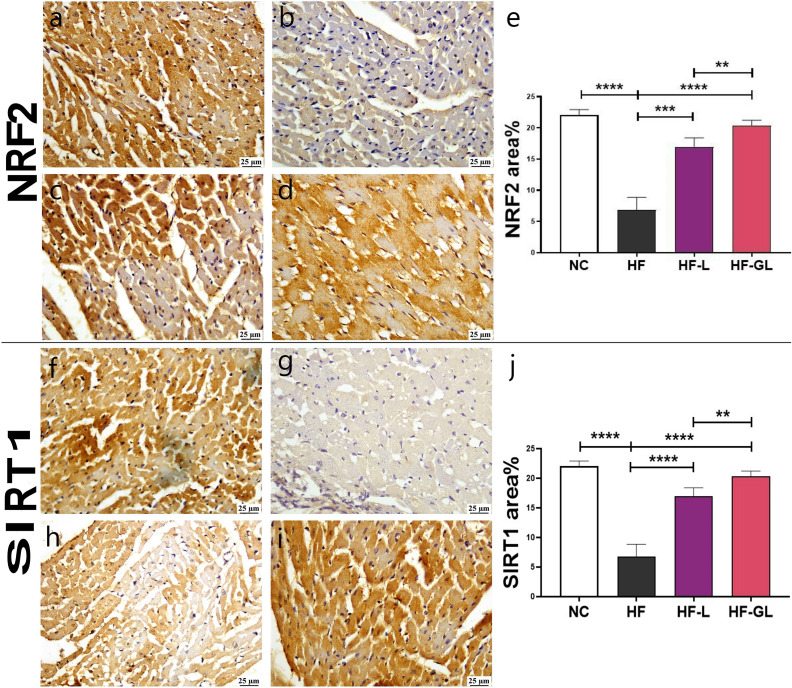
Immunohistochemistry of Nrf-2 and Sirt-1 expression in heart tissue in different groups; (a) marked expression of Nrf-2 in control group, (b) low expression of Nrf-2 in HF group, (c) moderate expression of Nrf-2 in the HF-L group, (d) high expression of Nrf-2 in the HF-GL group, (e) Charts present area % of Nrf-2 expression. (f) high expression of Sirt-1 in the control group, (g) mild expression of Sirt-1 in the HF group, (h) moderate expression of Sirt-1 in the HF-L group, (i) high expression of Sirt-1 in the HF-GL group, (j) Charts present area % of Sirt-1 expression. Data are expressed as means ± SE. Significant difference is considered at *P* < 0.05. (*) = summary of significance level.

The expression of NRF2 was significantly reduced in cardiomyocytes of the HF group compared to other groups. However, a significant increase in NRF2 expression was observed in the HF-L and HF-GL treated groups (Fig 3a-e).

Similarly, SIRT1 expression was markedly decreased in the HF group but significantly elevated in cardiomyocytes of all treated groups, with the highest expression recorded in the HF-GL group (Fig 3f-j).

## 4. Discussion

The rising prevalence of cardiometabolic diseases, driven by factors like excessive fructose consumption, highlights the urgent need for effective preventive agents. This study aimed to investigate the prophylactic cardioprotective effects of a combined extract from lemon and guava leaves—enriched with polyphenolic compounds—against high-fructose-induced cardiac injury. Our findings demonstrate that both individual lemon leaf extract and a combination of lemon-guava leaf extracts offer significant prevention of weight loss and metabolic dysregulation, restoration of cardiac biomarkers (CK, CK-MB, Troponin I), amelioration of oxidative stress (via increased GSH, GPx, SOD, CAT and reduced MDA), and suppression of pro-inflammatory cytokines (TNF-α, IL-6), and key cellular signaling pathways.

Plants are a fundamental natural source of numerous bioactive compounds [41]. Since ancient times, various edible plant preparations have been utilized in traditional medicine to treat numerous diseases. In contemporary times, the cosmetic, pharmaceutical, and nutraceutical industries have increasingly focused on plant-based formulations and pure phytochemicals. According to Tiwari et al, polyherbal remedies are rich in bioactive agents that act in synergistic, agonistic, or antagonistic manners and could achieve minimal side effects with therapeutic efficacy [42].

Our initial hypothesis postulated that the cardioprotective effects would be driven by the extracts’ rich polyphenolic content, a premise supported by our in vitro analyses. Our findings revealed a consistent trend, with lemon leaves exhibiting superior TPC and TFC, antioxidant activity, and enhanced effects in their combined formulation compared to guava leaves. The TPC of *C. limon* leaf extract aligned with prior studies, measuring slightly lower than South African samples (209.72 mg GAE/g) [43] but higher than Iraqi (148 mg GAE/g) and Syrian (128 mg GAE/g) variants [44,45]. Similarly, TFC corresponded with reported values for Syrian (103 mg QE/g DW) and Egyptian (137 mg QE/g DW) cultivars [45,46], though significantly lower than South African records (1052.58 mg GAE/g) [43]. Guava leaves demonstrated TPC and TFC values consistent with Thai (66 mg GAE/g) [47] and Indian (13.3 mg QE/g) [48] samples, yet these were lower than Korean TPC (150 mg GAE/g) [49] and Thai TFC (129 mg QE/g) [47]. These variations likely stem from genetic differences, environmental conditions [50], and cultivation practices [51].

Polyphenolic compounds serve as potent radical scavengers, underpinning the antioxidant capacity of plant extracts. Their phenolic groups enable diverse functions, including hydrogen donation, reduction reactions, and singlet oxygen neutralization [52]. In the DPPH assay, the stable free radical transitions from violet to yellow upon reduction by antioxidants, with the degree of color change quantifying radical-scavenging efficacy. This method is valued for its simplicity, speed, reproducibility, cost-effectiveness, and sensitivity [53]. Conversely, the FRAP assay measures antioxidant reducing power through the conversion of Fe^3^⁺ to Fe^2^ ⁺ , producing a spectrophotometrically detectable color shift proportional to antioxidant activity [54]. Employing both assays provides a comprehensive evaluation of antioxidant mechanisms, encompassing both radical neutralization and reducing capacity.

The current study identified a strong correlation between phenolic content (TPC, TFC) and antioxidant activity (DPPH, FRAP) in lemon, guava, and their composite extracts, corroborating earlier reports on phenolic-driven antioxidant effects [52]. Given the necessity of standardized natural extracts for reliable pharmacological research [55], we employed LC-MS/MS to quantitatively profile phenolic constituents in both leaves, ensuring precise metabolite characterization and methodological rigor. Our LC-MS/MS data concur with prior findings [46,56–61], while uniquely identifying dihydroxybenzoic acid in guava leaves and ellagic acid, chlorogenic acid, and hesperetin in lemon leaves—the latter two compounds having been previously documented in lemon fruits [62].

Herbs and polyherbal formulations have shown considerable potential in mitigating cardiovascular diseases associated with MetS by targeting multiple risk factors, including dyslipidemia, hypertension, and insulin resistance. Mechanistically, these natural products exert anti-inflammatory and antioxidant effects, contributing to the restoration of endothelial function and the reduction of atherosclerotic processes [63].

Our findings demonstrated a reduction in body weight gain and food consumption, accompanied by an increase in water intake in the high-fructose group. The prophylactic administration of either lemon leaf extract or the combination of lemon and guava leaf extracts significantly improved these parameters by effectively reducing fructose intake while increasing food consumption and weight gain. These results align with previous studies by Adoga et al. (2021) [64] and Sharma et al. (2022) [65], which reported a decrease in body weight in high-fructose-fed rats. The observed improvements in body weight and water intake in the treated groups may be attributed to enhanced insulin levels or sensitivity, leading to improved glycemic control, reduced fat catabolism in cells and tissues, and minimized muscle wasting [66]. These findings are consistent with studies indicating that polyphenol-rich plant extracts, such as those derived from lemon and guava leaves, ameliorate fructose-induced metabolic dysfunction [65,67]. The combined effect of the extracts is known to enhance insulin sensitivity and glucose metabolism [65].

The relative heart weight-to-body weight ratio was significantly elevated in the high-fructose group compared to the normal control group. This finding is consistent with previous research by Nwaneri-Chidozie et al. (2014) [68], which reported cardiac hypertrophy and myofibrillar degeneration. The prophylactic administration of lemon leaf extract or the combination of lemon and guava leaf extracts improved relative heart weight, likely due to their potent antioxidative and cardioprotective properties, as supported by earlier studies [68,69].

This study revealed that high fructose consumption led to elevated serum levels of cardiac function markers (CK, CK-MB, troponin I), blood glucose levels, insulin resistance, and dyslipidemia. Additionally, high fructose intake induced oxidative stress, characterized by increased MDA levels, reduced enzymatic and non-enzymatic antioxidants (GSH, GPx, SOD, CAT), and elevated pro-inflammatory cytokines (TNF-α and IL-6) in heart tissues. These findings are in agreement with recent studies by Wang et al. (2023) [70], Taskinen et al. (2019) [71], and Lê et al. (2009) [72], which demonstrated that high fructose intake contributes to metabolic syndrome, cardiac dysfunction, and oxidative stress.

Growing evidence suggests that excessive fructose consumption heightens the risk of cardiovascular diseases (CVD), contributing to dyslipidemia, inflammation, and coronary heart disease [73]. Increased intake of fructose-sweetened beverages is associated with a higher risk of CVD, partly due to fructose-induced obesity and insulin resistance [74]. However, the direct cardiotoxic effects of fructose are also a possibility [75]. Fructose-induced dyslipidemia is often accompanied by insulin resistance, which plays a crucial role in the development of metabolic syndrome [76]. Recent research further supports that individuals with elevated total cholesterol and low-density lipoprotein levels, coupled with reduced high-density lipoprotein levels, face a higher cardiovascular risk due to an imbalance between atherogenic and protective lipoproteins [77].

Elevated reactive oxygen species (ROS) levels and reduced antioxidant enzyme activity contribute to oxidative stress and cellular damage [78]. This study confirmed that high fructose intake induces oxidative stress, likely due to the impairment of the antioxidant defense system. Increased fructose catabolism may stimulate ROS production through lipid peroxidation [79]. Previous studies have indicated that diminished GPx activity results from radical-induced inactivation and enzyme glycation, whereas reduced SOD activity may be attributed to glycosylation or high hydrogen peroxide levels, which can impair its function [80,81]. This oxidative imbalance leads to systemic inflammation and increased pro-inflammatory cytokines [82], causing cardiomyocyte damage via ROS overproduction and lipid peroxidation, thereby elevating cardiac markers such as troponin I and CK-MB [83]. Wang et al. (2023) [70] similarly demonstrated that high-fructose-fed adult mice exhibited impaired cardiac function.

Hyperglycemia-induced ROS overproduction is a primary mechanism underlying diabetes-associated cardiomyocyte damage [84]. Prolonged hyperglycemia induces metabolic alterations that contribute to myocardial injury [85]. Given the roles of hyperglycemia, dyslipidemia, and oxidative stress in cardiac injury, citrus flavonoids have demonstrated cardioprotective effects through their antihyperglycemic, antihyperlipidemic, and antioxidant properties. Hesperidin has been shown to exert protective effects in ischemic heart disease in diabetic rats [86], while naringin has been reported to protect cardiomyocytes against hyperglycemia-induced injury in both in vitro and in vivo models [87].

Citrus flavonoids exhibit multiple health benefits, including antioxidant, anti-inflammatory, and cytoprotective effects [88]. Given the roles of oxidative stress and inflammation in diabetes and CVD [89], flavonoids’ antioxidant potential may significantly contribute to their therapeutic effects. Their chemical structure enables them to act as radical scavengers, oxygen quenchers, and hydrogen donors, thereby enhancing endogenous antioxidant defenses and preventing ROS-induced cellular damage [90].

Consistent with these findings, lemon and guava leaves have demonstrated promising prophylactic cardioprotective effects. Our results showed significant improvements in metabolic dysfunction and cardiac biomarkers in rats treated with lemon leaf extract or the combination of lemon and guava leaf extracts. The combination treatment showed more comprehensive preventive improvements than lemon extract alone across multiple parameters. Lemon leaves contain flavonoids that help lower blood pressure and cholesterol levels, essential for heart health, while guava leaves significantly reduce cardiac markers such as troponin I and CK-MB, thereby supporting heart function [91,92]. Both extracts regulate blood glucose levels, enhance insulin sensitivity, and effectively manage hyperglycemia [93]. The bioactive polyphenols and flavonoids in guava leaves, such as quercetin, ellagic acid, and gallic acid, contribute to their antidiabetic and antioxidative properties [94].

Our analysis revealed that lemon leaves are rich in citrus flavonoids, including rutin and naringenin. These flavonoids exhibit potent antioxidant properties, reducing ROS levels and enhancing antioxidant defenses, thereby mitigating lipid peroxidation and protein carbonylation in chronic diseases [95,96]. Furthermore, flavonoids prevent LDL oxidation, potentially reducing atherosclerosis risk [97].

Consistent with previous reports, polyherbal formulations often show enhanced efficacy compared to single herbs in treating various conditions, such as turmeric-ginger combinations for inflammation [98] and nigella-fenugreek-chicory-gymnema mixtures for cardiovascular disorders [99]. Our findings showed that the combination offers broader protection against fructose-induced cardiac dysfunction.

In this study, NRF2 expression was significantly increased in rats prophylactically administered with either lemon or the combination of guava-lemon leaf extracts compared to those fed a high-fructose diet. NRF2 plays a critical role in regulating inflammatory responses and maintaining cellular redox homeostasis [100]. Since excessive fructose consumption upregulates the production of TNF-α and IL-1β, exacerbating oxidative stress and accelerating cardiac inflammation [101], the activation or overexpression of NRF2 has been shown to mitigate heart injury by preventing cardiomyocyte damage, restoring mitochondrial function, and reducing oxidative stress [102]. These findings align with our histopathological observations of myocardial injury and inflammation, suggesting that NRF2 may offer unique cardioprotective benefits. Furthermore, our immunohistochemical analysis revealed that rats fed a high-fructose diet exhibited a significant reduction in SIRT1 expression, whereas rats prophylactically administered with lemon or guava-lemon leaf extracts showed elevated SIRT1 levels. SIRT1, a member of the sirtuin family, catalyzes the deacetylation of various substrates using nicotinamide and plays a central role in metabolic homeostasis [103]. Previous studies have demonstrated that SIRT1 overexpression protects cardiac myocytes from oxidative stress, inhibits apoptosis, and delays cardiac aging in mice [104,105], positioning it as another novel therapeutic approach for high-fructose-induced cardiac damage.

While this study provides valuable insights into the protective effects of guava-lemon leaf combinations against high-fructose-induced cardiac injury, some limitations should be acknowledged. First, the absence of a guava-only treatment group in the in vivo experiments prevents direct comparison of its individual efficacy with the combined extract, despite the in vitro evidence suggesting enhanced antioxidant activity in the mixture. Nevertheless, our findings highlight the potential of combined plant extracts as a prophylactic agent. The study was conducted on a rodent model, and further research is needed to validate these findings in human trials. Additionally, while we identified several key bioactive compounds, future studies should aim to isolate specific compounds to determine their individual contributions to the observed effects. Exploring the exact dose-dependent effects and long-term therapeutic potential beyond the prophylactic approach would also be valuable. Future studies focusing on the isolation and characterization of the individual active compounds are warranted to elucidate the precise mechanisms of action. Despite these limitations, this work lays important groundwork for developing plant-based interventions against metabolic stress-induced cardiac damage, offering a promising alternative to synthetic antioxidants.

## 5. Conclusion

Our *in vivo* findings demonstrate that the combined extract of lemon (*Citrus limon*) leaf and guava (*Psidium guajava*) leaf exert significant cardiometabolic protection in high-fructose-fed rats. The prophylactic benefits—including improved insulin sensitivity, normalized lipid profiles, and reduced oxidative stress and inflammation—were consistently more pronounced with the combined extract formulation. This combined action is likely mediated by the collective antioxidant and anti-inflammatory properties of the phytochemical constituents present in the extract. While the specific bioactive components responsible for this effect were not isolated in this study, our results provide a strong rationale for the traditional use of these plants and highlight the potential of the combined extract as a promising nutraceutical candidate.

## Supporting information

S1 Table The multiple reaction monitoring transitions and the optimized mass spectromer parameters. (PDF)

S1 Fig Calibration curve for standard gallic acid. (PDF)

S2 Fig Calibration curve for standard quercetin. (PDF)

S3 Fig The standard phenolics and flavonoids LC-ESI-MS/MS chromatograms in MRM mode. (PDF)

S4 Fig LC-ESI-MS/MS chromatograms in MRM mode of phenolics and flavonoids of (a) lemon leaf extract, (b) guava leaf extract. (PDF)

## Abbreviations

AI: Atherogenic index
AIN-93: American Institute of Nutrition 1993 (diet formulation)
CAT: Catalase
CK: Creatine kinase
CK-MB: Creatine kinase-myocardial band
CVD: Cardiovascular diseases
DPPH: 2,2-Diphenyl-1-picrylhydrazyl
ELISA: Enzyme-linked immunosorbent assay
FRAP: Ferric reducing antioxidant power
GAE: Gallic acid equivalents
GAE/g DW: Gallic acid equivalents per gram dry weight sample
GC-MS/MS: Gas chromatography-tandem mass spectrometry
GPx: Glutathione peroxidase
GSH: Reduced glutathione
HDL: High-density lipoprotein
HDL-Ch: High-density lipoprotein cholesterol
HOMA-IR: Homeostasis model assessment for insulin resistance
HWI: Heart weight index
IFCC: International Federation of Clinical Chemistry
IL-6: Interleukin-6
LC-ESI-MS/MS: Liquid chromatography–electrospray ionization–tandem mass spectrometry
LC-MS/MS: Liquid chromatography-tandem mass spectrometry
LDL: Low-density lipoprotein
LDL-Ch: Low-density lipoprotein cholesterol
MDA: Malondialdehyde
MetS: Metabolic syndrome
mM TE/g: Millimolar Trolox equivalents per gram
MRM: Multiple-reaction monitoring
NF-kB: Nuclear factor kappa B
NIH: National Institutes of Health
ROS: Reactive oxygen species
SOD: Superoxide dismutase
T2DM: Type 2 diabetes mellitus
T-Ch: Total cholesterol
TCA: Trichloroacetic acid
TF: Total flavonoid content
TFC: Total flavonoid content
TPC: Total phenolic content
TNF-α: Tumor necrosis factor-alpha
TG: Triglycerides
VLDL-Ch: Very low-density lipoprotein cholesterol
QE: Quercetin equivalents
QE/g DW: Quercetin equivalents per gram dry weight sample
DW: Dry weight
