# Supplementary material for: Combined polyphenols in Psidium guajava-citrus limon leaf extract attenuate fructose-induced cardiac injury by modulating metabolic and oxidative stress pathways in rats
Source: PLoS One. 2026 Jan 21;21(1):e0339641. doi: 10.1371/journal.pone.0339641 (PMC12822947; doi:10.1371/journal.pone.0339641)
Supplement: S1 Table — (PDF) [file pone.0339641.s001.pdf]

Table S1. The multiple reaction monitoring transitions and the optimized mass spectromer parameters.

Name	Q1 (m/z)	Q3 (m/z)	RT (min)	CE (V)	CXP (V)	DP (V)
Gallic acid	168.9	124.9	1.67	-30	-11	-110
	168.9	79	1.67	-30	-11	-110
Caffeic acid	178.9	135	5.83	-22	-9	-115
	178.9	107	5.83	-30	-7	-115
Rutin	609	299.9	9.13	-48	-15	-230
	609	270.9	9.13	-70	-9	-230
Coumaric acid	162.9	119	7.7	-20	-7	-90
	162.9	93	7.7	-40	-5	-90
Naringenin	271	151	20.98	-24	-25	-130
	271	119	20.98	-34	-11	-130
Querectin	301	151	18.16	-28	-9	-50
	301	178.8	18.16	-20	-7	-50
Ellagic acid	301	145	8.97	-40	-14	-120
	301	245	8.97	-38	-14	-120
3,4-Dihydroxybenzoic acid	152.9	109	3.13	-40	-5	-75
	152.9	90.9	3.13	-20	-7	-75
Hesperetin	301	164	22.62	-23	-10	-125
	301	136	22.62	-38	-10	-125
Cinnamic acid	146.9	102.6	18.16	-17	-6	-60
	146.9	77	18.16	-33	-6	-60
Methyl gallate	183	124	5.04	-30	-10	-110
	183	140	5.04	-30	-10	-110
Kaempferol	284.7	93	22.08	-46	-10	-120
	284.7	116.8	22.08	-52	-10	-120
Ferulic acid	192.8	133.9	8.89	-16	-5	-25
	192.8	177.9	8.89	-12	-5	-25
Syringic acid	196.9	122.8	6.23	-24	-5	-30
	196.9	181.9	6.23	-12	-5	-30
Apigenin	269	151	21.47	-15	-7	-35
	269	117	21.47	-15	-7	-35
Catechin	288.8	244.9	5.05	-16	-8	-40
	288.8	109	5.05	-32	-8	-40
Daidzein	253	132	16.22	-55	-10	-65
	253	91	16.22	-50	-13	-65
Chlorogenic acid	353	191	5.1	-23	-10	-60
	353	179	5.1	-35	-10	-60
Resveratrol	227.0	185	14.27	-26	-10	-53

	227.0	143	14.27	-40	-10	-53
Rosmarinic acid	359.1	161	13.42	-20	-10	-60
	359.1	197	13.42	-20	-10	-60

CE, Collision energy; CXP, Collision cell exit potential; DP, Declustering potential.
