# Supplementary figures and images for: Combined polyphenols in Psidium guajava-citrus limon leaf extract attenuate fructose-induced cardiac injury by modulating metabolic and oxidative stress pathways in rats
Source: PLoS One. 2026 Jan 21;21(1):e0339641. doi: 10.1371/journal.pone.0339641 (PMC12822947; doi:10.1371/journal.pone.0339641)

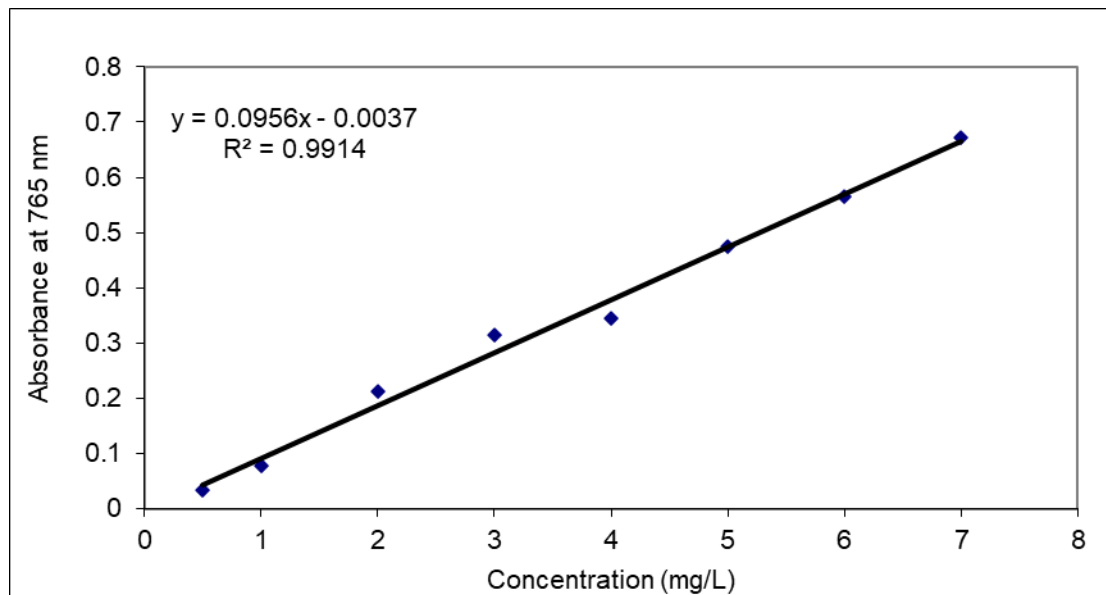

**Suppl. Figure S1. Calibration curve for standard gallic acid,**

Supplement: S1 Fig — (PDF) [file pone.0339641.s002.pdf]

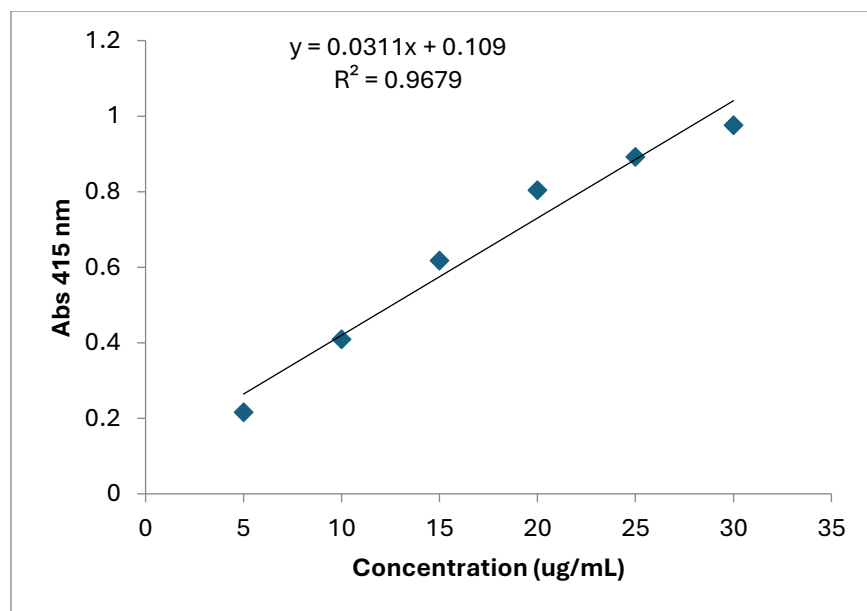

**Suppl. Figure S2. Calibration curve for standard quercetin**

Supplement: S2 Fig — (PDF) [file pone.0339641.s003.pdf]

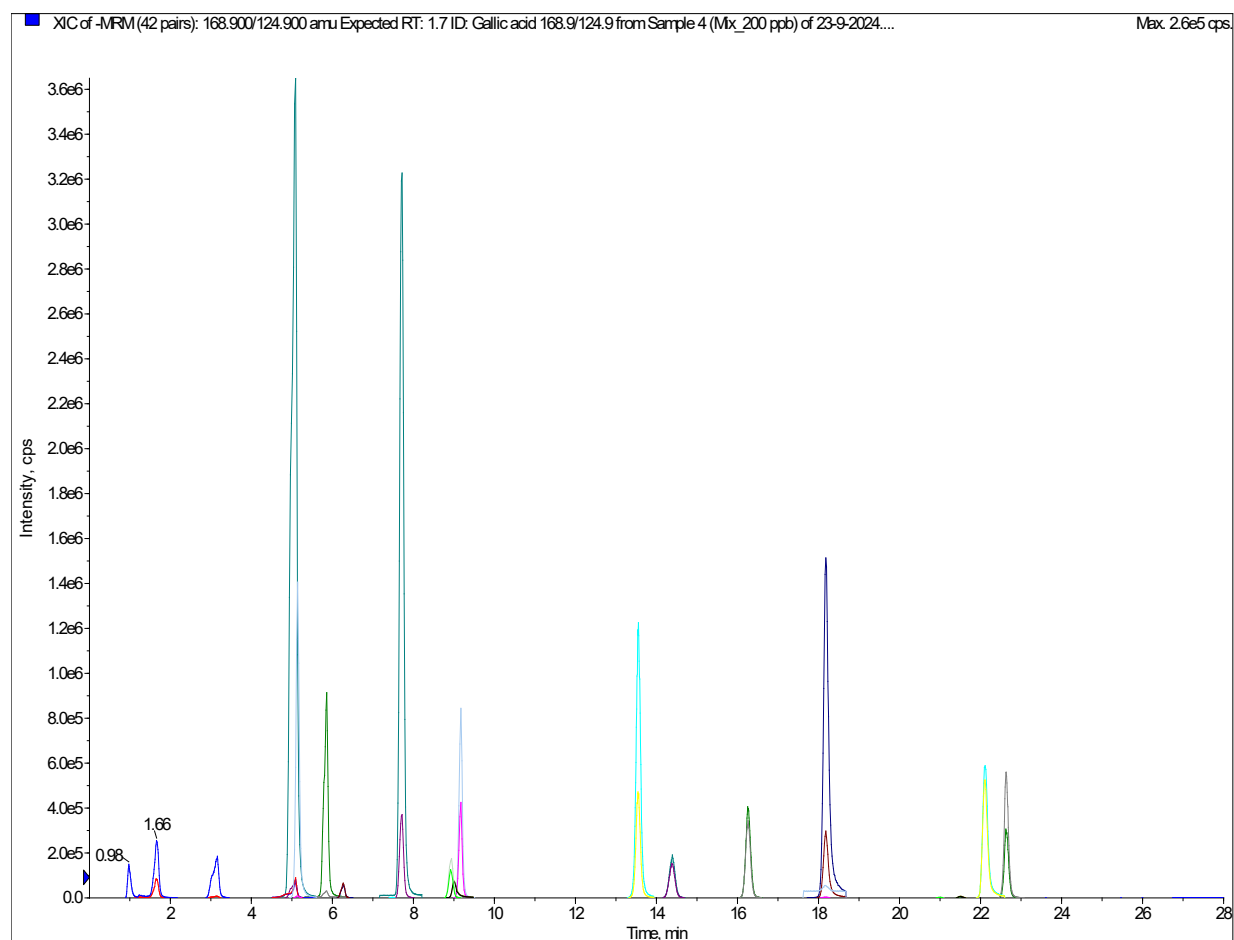

**Suppl. Figure S3. The standard phenolics and flavonoids LC-ESI-MS/MS chromatograms in MRM mode**

Supplement: S3 Fig — (PDF) [file pone.0339641.s004.pdf]
