# Supplementary material for: Combined polyphenols in Psidium guajava-citrus limon leaf extract attenuate fructose-induced cardiac injury by modulating metabolic and oxidative stress pathways in rats
Source: PLoS One. 2026 Jan 21;21(1):e0339641. doi: 10.1371/journal.pone.0339641 (PMC12822947; doi:10.1371/journal.pone.0339641)
Supplement: S4 Fig — (PDF) [file pone.0339641.s005.pdf]

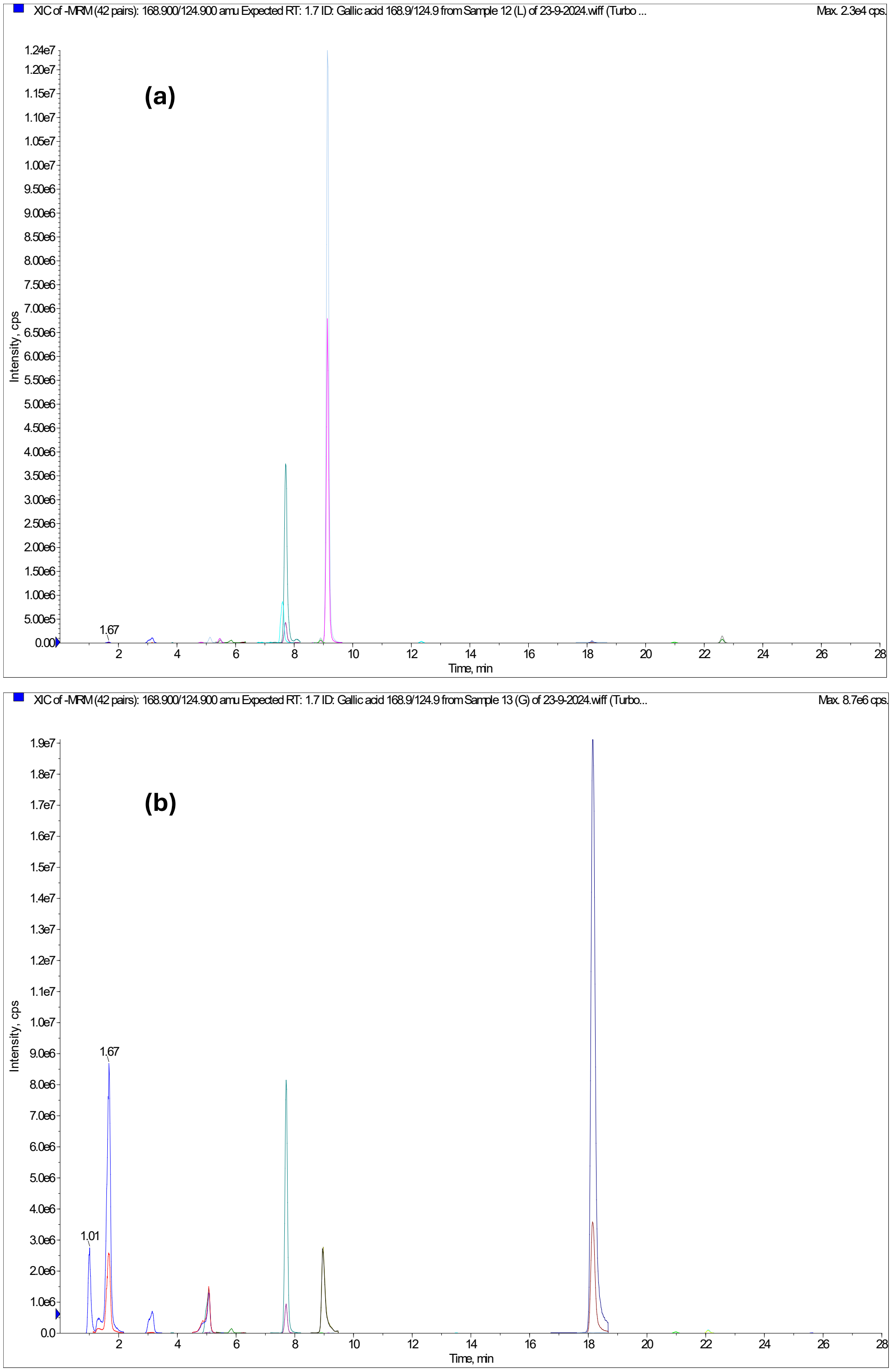

Suppl. Figure S4 . LC-ESI-MS/MS chromatograms in MRM mode of phenolics and flavonoids of (a) lemon leaf extract, (b) guava leaf extract.
